# Morphological, molecular and phytochemical variations induced by colchicine and EMS chemical mutagens in *Crocus sativus* L.

**DOI:** 10.1016/j.fochms.2022.100086

**Published:** 2022-02-14

**Authors:** Negin Samadi, Mohammad Reza Naghavi, Natalia Moratalla-López, Gonzalo L. Alonso, Majid Shokrpour

**Affiliations:** a Department of Horticultural Science, College of Agriculture and Natural Resources, University of Tehran, 31587-77871 Karaj, Iran; b Department of Agronomy and Plant Breeding, College of Agriculture and Natural Resources, University of Tehran, Karaj, Iran; c Cátedra de Química Agrícola, ETSI Agrónomos y Montes, Universidad de Castilla-La Mancha, Campus Universitario, 02071 Albacete, Spain

**Keywords:** Crocetin esters, *Crocus sativus*, Flow cytometry, Picrocrocin, Relative gene expression, Safranal

## Abstract

*Crocus sativus* L., also known as saffron, is one of the most important medicinal and spice plants throughout the world. The plant is a rich source of apocarotenoids such as crocetin esters, picrocrocin, and safranal. The purpose of this study was to investigate the effect of colchicine and ethyl methanesulfonate (EMS) mutagens on possible inducing new variation in *C. sativus.* Accordingly, corms were exposed to EMS (0.1% and 0.2%) and colchicine (0.05% and 0.025%) for three incubation times. The lowest survival rate of corms was related to EMS treatments. The relative expression of *ALDH*, *BGL*, and *CCD2* genes under 0.025% colchicine treatment for 12 h revealed a 2 fold increase compared with the control. The flow cytometric measurements (FCM) of the nuclear DNA content of the colchicine-treated plants did not reveal any significant changes in 2C DNA content. The results, manifest the potential of mutagens to create new variations in the plant.

## Highlights

• The highest expression level of the *ALDH*, *BGL*, and *CCD2* genes was found in 0.025% colchicine for 12 h treatment.
• The content of crocetin esters, picrocrocin, and safranal in colchicine treatments was changed.
• The stigmas of the *C. sativus* flowers with two, four, five, and six threads were observed.
• Various differences in morphological traits were observed in both colchicine and ethyl methanesulfonate (EMS) treatments.
• The lowest survival rate of corms was related to ethyl methanesulfonate (EMS) treatments.

## Graphical abstract

**Figure:**
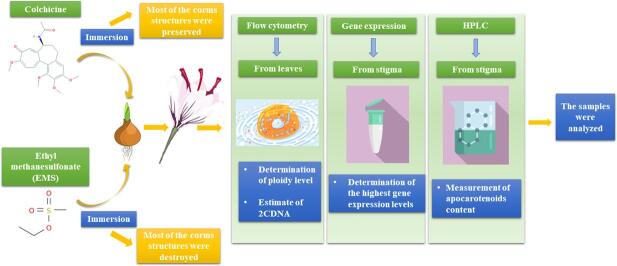

## 1 Introduction

*Crocus sativus* L. is a well-known plant species with tremendous medicinal apocarotenoids such as crocetin esters (also known as crocins), picrocrocin, and safranal. These crucial compounds are produced and reserved in the tissue of the flower stigmas, which is known as saffron (dried stigmas of Crocus flower) (Wani et al., 2017). These apocarotenoids are derived from primary metabolites, and give the specific aroma, taste, and colour to the *C. sativus* (Parizad et al., 2019). In general, one of the main characteristics of *C. sativus* is its thread-like red stigmas (Yue et al., 2020) (Supplementary File 1. Figure S1A).

The accumulating of apocarotenoids at the red stage of stigma is catalysed by the oxidative cleavage of zeaxanthin through carotenoid cleavage dioxygenase (CCD2). Then, the 3-hydroxy-cyclocitral and crocetin dialdehyde undergo dehydrogenation and glucosylation to make crocin and picrocrocin. Finally, after harvesting, when stigma is dried, picrocrocin will produce safranal through the combined activity of heat and β-glucosidase (Ahrazem et al., 2016, Frusciante et al., 2014, Jain et al., 2016). It is worth noting that there is an alternative pathway where the inter-conversion of zeaxanthin and violaxanthin occur by zeaxanthin epoxidase (ZEP) and violaxanthin de-epoxidase (VDE), depending on the light intensity (Jain et al., 2016) (Supplementary File 1. Figure S1B).

*C. sativus* is a valuable medicinal plant due to its medicinal apocarotenoids. The secondary metabolites represent the potential useful properties of the plant, including attributes that can treat diverse diseases such as cardiovascular diseases, anxiety, depression, and nervous disorders as well as different types of cancers (Jain et al., 2016).

*C. sativus* is a perennial and sterile triploid plant of the Iridaceae family (Jain et al., 2016). Iran, which produces nearly 90% of the world's saffron, is the primary producer of this plant all over the world (Parizad et al., 2019). *C. sativus* grows in temperate climates across the world, from east Asia (especially in Iran) to west Europe and Spain, Greece, India, Italy, Azerbaijan, and Morocco (Shokrpour, 2019).

*C. sativus* has three homologous sets of chromosomes (2n = 3x = 24), and its closest relative is assumed to be a diploid ancestor of *C. carthwrightianus*, because *C. sativus* is probably the result of a fortuitous mutant. Also, since some Minoan frescos in the southern Aegean islands of Crete and Santorini have documented the use of *C. sativus* in around 700 BCE, *C. sativus* probably comes from a Greek origin (Nemati et al., 2019, Yue et al., 2020).

The main factor that hampers *C. sativus* genetic improvement is the triploid genetic features which are maintained by its corm vegetative growth (Yue et al., 2020). However, there is only one well-known cultivar of *C. sativus* around the world, and the real genetic variation of this plant worldwide is currently unknown (Agayev, Fernandez, & Zarifi, 2009).

The breeding of *C. sativus* as a sterile plant is completely distinct from other plants. Due to its complexity and extraordinary characteristics, diverse strategies have been presented to improve the product quality and quantity. Research has reported divergent results about the existence or non-existence of genotypic variation in *C. sativus*, and there is no consensus on this issue (Busconi et al., 2018, Grilli Caiola et al., 2001).

Clonal selection, as a classical approach, might have promising results. It is one of the different methods used in the *C. sativus* breeding programs to cultivate high-yielding cultivars. However, it has been shown that clonal selection in *C. sativus* is like an enigma that has yet to be solved. Some researchers have proposed contradictory beliefs about the use of clonal selection. They argue that as a cloned species, there is probably insufficient genetic diversity to implement *C. sativus* breeding programs (Agayev et al., 2009).

Further, there are several other important processes involved in the genetic diversity of plant species such as hybridization, recombination, and mutation (Donini & Sonnino, 1998). It is impossible to use plant hybridization that causes recombination events in *C. sativus* due to the sterile pollen production. Also, the rate of natural mutations is generally low in the plant genomes. One of the useful methods that can improve crops is induced mutation (Donini and Sonnino, 1998, Shokrpour, 2019). The goal of induced mutation in *C. sativus* breeding programs is to develop unique traits at chromosomal and gene levels, so that it could be considered as an alternative tool for *C. sativus,* which is propagated vegetatively by corm in order to induce desirable and effective genetic diversity on quantitative and qualitative features (Shokrpour, 2019).

The potential of induced mutations in the crop genetic improvement has already been shown and this method can efficiently enhance natural genetic resources. Induced mutations play a key role in enhancing food security worldwide, as creating new types of food crops has contributed to the growth of crop production (Kharkwal and Shu, 2009, Rime et al., 2019). The induced mutations, generated by physical (radiation) or chemical mutagens, make random changes in nuclear or cytoplasmic DNA. They produce different changes such as chromosomal or genomic mutations (e.g. deletions, insertion, translocations, duplications, etc.) that lead to variability (Donini and Sonnino, 1998, Shu et al., 2012).

The effects of physical or chemical mutagens are distinct. That is, it has been shown that physical mutagens induced have more deleterious effects as the frequency of recessive mutations is higher than dominant mutations (Shu et al., 2012). On the other hand, using physical mutagens such as X-ray, γ-ray, or other physical components requires specialized and advanced equipment that are difficult to obtain (Chen et al., 2018). On the contrary, chemical mutagens provoke single nucleotide polymorphisms (SNPs), and functional mutations induced at the genome level are more likely to be dominant or co-dominant. The probable changes can become a source of genetic diversity in various products (Shu et al., 2012).

Many chemical mutagens are used in plant breeding programs. Ethyl Methane Sulfonate (EMS) is one of the most common chemical mutations widely used to induce point mutation in DNA (Rime et al., 2019). EMS, as an alkylating agent, leads to the transformation of C nucleotide into T nucleotide, after which C/G converts into T/A (Serrat et al., 2014).

Colchicine is another mutagen that is used both for polyploidy induction in plants and mutation induction. The mutagen, as a bioactive alkaloid and a poisonous compound, is extracted from seeds and corms of the meadow saffron (*Colchicum autumnal* L.) (Sattler, Carvalho, & Clarindo, 2016). The main mechanism of colchicine is binding with alpha- and beta-tubulin dimers, which inhibits microtubule polymerization during the cell cycle (mitosis) in plant cells, after which chromosomes/chromatids migration is halted during the anaphase stage. It is acknowledged that cell division is blocked by colchicine mutagen, but its accurate mechanism in chromosomes and polyploidy induction of plants is still uncertain (Manzoor et al., 2019, Sattler et al., 2016, Zhou et al., 2017). It is worth noting that the complex genome of *C. sativus* is sizable, estimated to be about 10.5 Gbases (Busconi et al., 2018, Jain et al., 2016).

Zaffar, Wani, Anjum, and Zeerak (2003) studied the induction of changes in *C. sativus* by colchicine under four different aqueous concentrations. They reported delayed flowering and leaf emergence, flowers with serrated and irregular shaped tepal and flowers with deep-red pigmentation in stigmas. Khan, Nagoo, Naseer, Nehvi, and Zargar (2011) used EMS and colchicine to improve corms propagation in *C. sativus*, with their results demonstrating the larger number of daughter corms per mother corm by 0.1% EMS and 0.05% colchicine treatments. Ali et al. (2012) studied daughter corm production at different concentrations of colchicine, reporting that the best treatments for raising the number of daughter corms were attained at doses of 0.25% and 0.50% colchicine.

Previous studies have solely focused on morphological characteristics in *C. sativus*, and the lack of research on the analysis of gene expression or phytochemical contents is felt. Hence, the aim of this study was to investigate the changes in morphological features and genome size of the *C. sativus* plants due to the effects of mutagens. It was intended to assess variations in the expression of *ALDH*, *BGL*, and *CCD2* genes and to determine the crocetin esters, picrocrocin, and safranal contents.

## 2 Materials and methods

The *C. sativus* corms were collected from a farm in Neyshabur, Iran (36°, 12′, 59″). For this purpose, we gathered corms weighing 18 ± 2 g and removed their crown fibres cover completely. The corms were treated with aqueous solutions of colchicine)0.025% and 0.05%(and EMS (0.1% and 0.2%) for three incubation times, 12 h and 24 h continuously, and 24 h discontinuously (dc) (i.e. during 24 h, the corms were first immersed in the mutagens and then removed after 12 h. Finally, they were soaked again in the mutagens solutions for another 12 h.) at room temperature. The control corms were incubated with the distilled water with about 40 corms in each treatment. The type of mutagens, their concentrations and incubation times were calculated from previous studies (Khan et al., 2011, Zaffar et al., 2003). All the treatments were applied in the 16th of September 2017 when the buds were not dormant because based on the Córcoles, Ramos, García, Valverde, and Riquelme (2015) in this phenological stage, the buds were starting to swelling and sprout. Following the treatment of corms with mutagens, they were sowed at rows 30 cm apart at 25 cm intervals with a cultivation depth of 15–20 cm at the research farm of the Department of Horticultural Science and Landscape Engineering, University of Tehran Located in Mohammadshahr research station, Karaj, with a latitude of 36 degrees and 19 min north and a longitude of 59 degrees east and 38 min north and an altitude of 1320 m above sea level (Karaj- Iran).

### 2.1 Measurement of morphological parameters

In the flower of *C. sativus*, the undifferentiated petals and sepals are called tepals. Hence, its flowers have six tepals, three yellow stamens, and a white filiform style culminated in a red stigma that is divided into three threads. To assess the effect of colchicine and EMS mutagens on the plant growth traits, morphological parameters were measured from sprouting to flowering stages. The traits consisted of corm survival rate, corm sprouting time, first flowering time, days to 50% flowering, stigma length and diameter, number of flowers, and flowering duration (from the first flowering to the end of flowering).

### 2.2 Gene expression analysis

The total mRNA was extracted from 100 mg red stigma tissue in all bulked stigma samples (16 stigmas were used for each replicate) using DENAzist Column RNA Isolation Kit (DENAzist Asia Co., S-1010, Mashhad, Iran), according to the manufacturer’s instructions. The extracted RNA was purified by Thermo Scientific DNase I kit (Fermentas, EN0521, Thermo Fisher Scientific, Hudson, NH, USA) to remove remaining genomic DNA contamination in accordance with the manufacturer’s instructions. The quality and quantity of mRNA were determined by NanodropTM 2000c spectrophotometer (Thermo Scientific, USA) and its integrity was checked by agarose gel electrophoresis (%1) (Supplementary File 1. Figure S2). The cDNA was synthesized by 1 μg of total RNA using Takara qPrimeScript™ RT reagent Kit (TAKARA, RR037A, Kusatsu, Shiga, Japan) based on the manufacturer’s instructions to obtain a 20 μL cDNA solution. The qPCR primers were designed by the Primer Quest software and then checked with oligo analyzer tool (eu.idtdna.com/calc/analyzer) and NCBI/Primer-BLAST (http://www.ncbi.nlm.nih.gov/tools/primer blast/index.cgi? LINK_LOC=BlastHome). According to the Ahrazem et al. (2016), the *18S* rRNA gene was used as the reference gene (Supplementary File 2. Table S1). The qPCR was performed using specific primers (Supplementary File 2. Table S1) on an ABI StepOneTM Real-Time PCR system (Applied Biosystems, California, USA) with the 5x HOT FIREPol® EvaGreen® qPCR Mix Plus ROX (Solis BioDyne, 08–24-0000S, Estona) according to the manufacturer’s instructions. 1 μL of the first-strand cDNA was used as a template in 20 μL reactions, including three μL EvaGreen qPCR Mix Plus and five pmol of each primer. The efficiency of all primer pairs with serial dilutions of cDNA was calculated from the following formula: E = 10^−1/slope^ − 1. In qPCR, this value was in the range of 85.89 to 120.49 and R2 (the coefficient of determination) was greater than 0.98. The qPCR was run at 95 °C for 15 min, 40 cycles at 95 °C (15 s), 57 °C (20 s), and 72 °C (20 s), followed by a gradient at 60–95 °C (1 min). To perform qPCR, three biological replicates for each sample and two technical replicates for each biological sample were used. In all the qPCR tests, the negative control of Master Mix was implemented in addition to primers. The specificity of qPCR products was determined by the fluorescence data. The amplification curve of each primer pairs was sigmoidal, and the melting curve indicated just one peak, relevant to the specific product of each gene with a specific melting point (Supplementary File 1. Figure S3). The relative gene expression levels were computed using the Pfaffl method (Pfaffl, 2012). It is worth mentioning that most of EMS-treated plants disappeared after exposure and thus excluded from the assessment.

### 2.3 Phytochemical assay

The content of three important apocarotenoids including crocetin esters, picrocrocin, and safranal was assayed by HPLC–DAD in the University of Castilla-La Mancha, Spain. In this method, the phytochemical analysis of each sample requires at least 1 g of stigma powder, but due to the small number and lightweight of stigmas, the sample was insufficient; hence, treatment replicates were pooled for each treatment. The harvested stigmas were dried according to ISO 3632 (2011) standard. The apocarotenoids were extracted from saffron (dried stigma) according to the method introduced by García-Rodríguez et al. (2014). Colchicine-treated plants were just measured as described earlier.

#### 2.3.1 Preparing saffron extract

The aqueous extracts of saffron were prepared according to ISO 3632 (2011) standard. About 500 mg of powdered saffron (dried stigma), previously passed through a 0.5 mm mesh sieve, was transferred to a 1 L volumetric flask before adding 900 mL of Milli-Q water. The solution was stirred by a magnetic stir at 1000 rpm for 1 h under dark condition. The solution was made up to 1 L, and then filtered by a filter made from Hydrophilic Polytetrafluoroethylene (PTFE) equipped with a 0.45 μm mesh (Millipore, Bedford, MA). Afterwards, it was transferred into a vial for HPLC–DAD analysis. The saffron extraction was re-checked by performing a second extraction through the filtration of the first extraction based on ISO 3632 (2011) standard (Garcia-Rodriguez et al., 2014).

#### 2.3.2 HPLC-DAD analysis

Overall, 20 µL of saffron aqueous extract of each sample was injected into an Agilent 1200 HPLC chromatograph (Palo Alto, CA, USA) with an inner diameter of 150 × 4.6 mm, 5 µm Teknokroma (San Cugat del Vallès, Barcelona, Spain) Brisa LC2 C_18_ column, which was equilibrated at 30 °C. The eluents comprised water (A) and acetonitrile (B) with the following gradient: 20% B, 0–5 min; 20–80% B, 5–15 min; and 80% B, 15–20 min with a flow rate of 0.8 mL/min. The DAD detector (Hewlett-Packard, Waldbronn, Germany) was set at 250, 330, and 440 nm for picrocrocin, safranal, and crocetin esters, respectively. The analysis, identification, and quantification of the picrocrocin, safranal and crocetin esters were conducted based on saffron aqueous extract using the method described by Garcia-Rodriguez et al. (2014). All analyses were performed in duplicate and two measurements were performed for each replicate.

#### 2.3.3 UV–vis analysis

The colouring strength ($E_{1cm}^{1\%}$ 440 nm) value of each saffron extract was determined according to ISO 3632 (2011) by a Perkin-Elmer Lambda 25 spectrophotometer (Norwalk, CT, USA) using UV Win Lab 2.85.04 software (Perkin-Elmer). In this research, all the analyses were performed in duplicate and two measurements were conducted for each replicate.

### 2.4 Ploidy level and genome-size determination

The flow cytometric measurements (FCM) was conducted using propidium iodide (PI) staining technique and *Vicia faba* cv. Inovec (2C DNA = 26.90 pg,) (Doležel, Greilhuber, & Suda, 2007) as an internal reference for a standard plant in order to estimate the ploidy level of the colchicine-treated plants, which had a different appearance in the first and second years of the experiment. 15 cm of *C. sativus* leaves and 2 cm of *V.faba* cv. Inovec leaves were chopped with a sharp razor blade in a petri dish containing 2 mL of woody plant buffer (WPB) (Loureiro, Rodriguez, Doležel, & Santos, 2007), after which the nuclei suspension was filtered using a Partec (Partec, Münster, Germany) with a nylon mesh of 30 μm. Then, 80 μL of RNase and 80 μL of PI were added to the nuclei suspension. The nuclei suspension was analysed by BD FACSCantoTM-KE flow cytometer (BD Biosciences, Bedford, MA, USA) equipped with an Argon ion laser (488 nm) using BD FACSDivaTM software to determine the amount of genomic 2C DNA. Besides, three replicates were used in each treatment for genome size measurement. Histograms were gated using Partec (Partec, Münster, Germany) FloMax ver. 2.4e. The relative fluorescence intensity of stained nuclei was measured on a linear scale. The absolute DNA content of a sample was estimated based on the values of G1 peak means (Doležel et al., 2007, Dolezel, 2003) as follows:

(1) $$\text{Sample}\;2\text{C DNA}\;\left(\text{pg}\right)\;\text{content}=\left(\text{sample G}1\;\text{peak mean}/\text{standard G}1\;\text{peak mean}\right)\times \text{standard}\;2\text{C DNA amount}\;\left(\text{pg}\right).$$

Where 1 pg of DNA denotes 978 mega base pairs (Mbp) (Dolezel, 2003).

### 2.5 Data analysis

The experiment was run using the Randomized Complete Block Design (RCBD) with four replications. The raw data were analysed using IBM SPSS 22 software (SPSS, 2013). The comparison of means was carried out using LSD at a probability level of 0.01. The discrepancies of control and treatment groups were compared using Dunnett at a probability level of 0.05. Furthermore, the standard error (SE) was computed.

## 3 Results

### 3.1 Mutagens and survival rate

Colchicine- and EMS- treated plants had different morphological traits. Also, changes in the morphology of treated samples were different than the controls. Moreover, colchicine mutagen had no significant effect on the corms survival rate compared with the controls, but its survival rate was higher. By contrast, EMS mutagen caused a considerable change in the survival rate (Table 1).

**Table 1:** Effect of colchicine and EMS on corms of *C.sativus* survival rate (%)

Survival rate (%)													
0.025%			0.05%			Control%	0.1%			0.2%			Control%
24 h	24 h dc	12 h	12 h	24 h dc	12 h	0.00	24 h	24 h dc	12 h	24 h	24 h dc	12 h	0.00
100^ns^	95^ns^	95^ns^	92.5^ns^	97.5^ns^	80^ns^	88.33	32.50^ns^	30^ns^	60^ns^	10*	17.5*	25*	83.33

ns and * representing non-significant and significant differences, respectively (Dunnett; P ≤ 0.05). Exposing *C.sativus* corms to different concentrations of colchicine (0.05 and 0.025%) and EMS (0.1 and 0.2%) for 12 h and 24 h continuously, and 24 h discontinuously (dc).

### 3.2 Physiological and morphological traits

Treatments with 0.05% and 0.025% colchicine for 24 h dc led to earlier corm sprouting. Also, different concentrations of colchicine had a significant effect on the first flowering time compared with the control. Moreover, treatments with 0.025% colchicine for 24 h, 24 h dc, and 12 h increased the number of days before the end of flowering (flowering duration). Also, treatment with 0.025% colchicine for 12 h expanded the stigmas diameter. The number of days to reach 50% flowering was higher in all colchicine-treated samples. However, this chemical mutagen had no significant effect on the number of flowers and stigma length characteristics (Table 2).

**Table 2:** Effect of colchicine on Morphological and Growth traits of *C.sativus*

Traits	Colchicine						
	0.025%			0.05%			0.00%
	24 h	24 h dc	12 h	24 h	24 h dc	12 h	Control
Corm sprouting time (day)	23^ns^	21*	24 ^ns^	23 ^ns^	21*	27 ^ns^	24
First flowering time (day)	14*	14*	15*	14*	14*	14*	11
Flowering duration (day)	19*	19*	20*	18^ns^	18^ns^	18^ns^	17
No. of flower	4.79^ns^	5.18^ns^	4.36^ns^	4.79^ns^	3.97^ns^	5.50^ns^	4.35
Stigma length (mm)	27.77^ns^	27.84^ns^	28.11^ns^	26.91^ns^	27.46^ns^	28.01^ns^	27.18
Stigma diameter (mm)	0.677^ns^	0.662^ns^	0.663^ns^	0.676^ns^	0.671^ns^	0.751*	0.633
50% Flowering time (day)	15*	14*	17*	14*	14*	15*	11

Values are the means of 40 biological replicates. ns and * representing non-significant and significant differences, respectively (Dunnett; P ≤ 0.05). *C. sativus* plants following exposure to colchicine mutagen at different concentrations (0.05 and 0.025%) for 12 h and 24 h continuously, and 24 h discontinuously (dc).

The number of days until the first flowering time and the number of days to reach 50% flowering spiked in all EMS treatments compared with the control, except for 0.1% EMS for 24 h. Nevertheless, it was the only treatment in which the treated plants’ flowering period was shorter than that of controls. The average number of flowers dropped significantly after the treatment with EMS, but it had no significant effects on the length and diameter of stigmas and the sprouting time (Table 3).

**Table 3:** Effect of EMS on Morphological and Growth traits of *C.sativus*

Traits	EMS						
	0.1%			0.2%			0.00%
	24 h	24 h dc	12 h	24 h	24 h dc	12 h	Control
Corm sprouting time (day)	26^ns^	36^ns^	29 ^ns^	40 ^ns^	24^ns^	50^ns^	26
First flowering time (day)	9^ns^	13*	12*	11*	12*	13*	8
Flowering duration (day)	9*	13^ns^	12^ns^	13^ns^	12 ^ns^	14^ns^	14
No. of flower	1*	1*	1.5*	1.5*	1.75*	2*	4.12
Stigma length (mm)	29.81^ns^	25.55^ns^	26.10^ns^	26^ns^	26.95^ns^	26.60^ns^	26.45
Stigma diameter (mm)	0.770^ns^	0.690^ns^	0.630^ns^	0.770^ns^	0.645^ns^	0.660^ns^	0.601
50% Flowering time (day)	9^ns^	14*	13*	14*	13*	14*	9

Values are the means of 40 biological replicates. ns and * representing non-significant and significant differences, respectively (Dunnett; P ≤ 0.05). *C. sativus* plants following exposure to EMS mutagen at different concentrations (0.1 and 0.2%) for 12 h and 24 h continuously, and 24 h discontinuously (dc).

### 3.3 Identification of colchicine- and EMS- treated plants characteristics

**Fig. 1:**
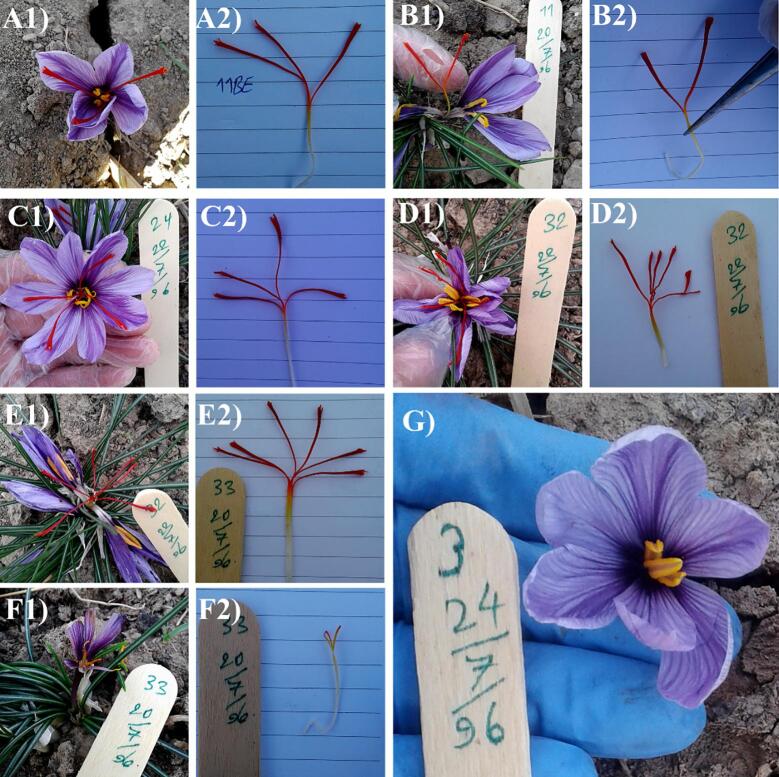
Effect of colchicine on flowers shapes and stigma of *C. sativus*. Control (A), two-thread stigma with six tepals (B), four-thread stigma with eight tepals (C), five-thread stigma with ten teplas (D), six-thread stigma with ten teplas (E), deformed flower with incomplete stigma and tepals (F), and flower without sigma (G).

After the growth period, some colchicine-treated plants exhibited morphological modifications in the first and second years of cultivation. It is worth mentioning that in the first growing season, the number of cultivated plants was lower than the second year, and there were only a few mutagen-treated plants that produced flowers without tepals and stigma (Supplementary File 1. Figure S4). It was followed by some plants generating flowers with varying numbers of stigmas, such as two, four, five, and six threads in the second growing season. Besides, flowers without stigma and deformed flowers with incomplete tepals and stigmas were observed in the second cultivation year (Fig. 1).

The EMS treatments considerably decreased the percentage of corms sprouting. Therefore, in order to identify changes, the non-sprouting corms were removed from the ground. Changes in EMS-treated plants, including the disintegrated and crushed corms, were more significant, destroying most of the corm structures (Supplementary File 1. Figure S5). Furthermore, EMS mutagen exhibited abnormalities in the treated plants, such as lack of asymmetry in tepals, irregularly shaped flowers, incomplete stigma, and angled tepals (Supplementary File 1. Figure S6).

### 3.4 Flow cytometry analysis and genome size measurement

Among colchicine-treated *C. sativus* plants, the value of 2C DNA ranged from 10.6 pg/2C (at 0.025% colchicine for 24 h dc) to 11.30 pg/2C (at 0.025% colchicine for 24 h) (Supplementary File 2. Table S2), and there was no significant difference between plants in terms of the nuclear 2C DNA. The histograms employed for the analysis of nuclear DNA content comprised two peaks: peak 1 indicates the G1 of an unknown *C. sativus* species and peak 2 represents the G1 of a known *Vicia faba* cv. Inovec (2C DNA = 26.90 pg) as the internal reference standard (Supplementary File 1. Figure S7). The coefficient of variation (CV) of G1 peaks for *C. sativus* species and *V. faba* samples was less than 5%.

### 3.5 *Relative expression analysis of ALDH*, *BGL*, and *CCD2 genes*

**Fig. 2:**
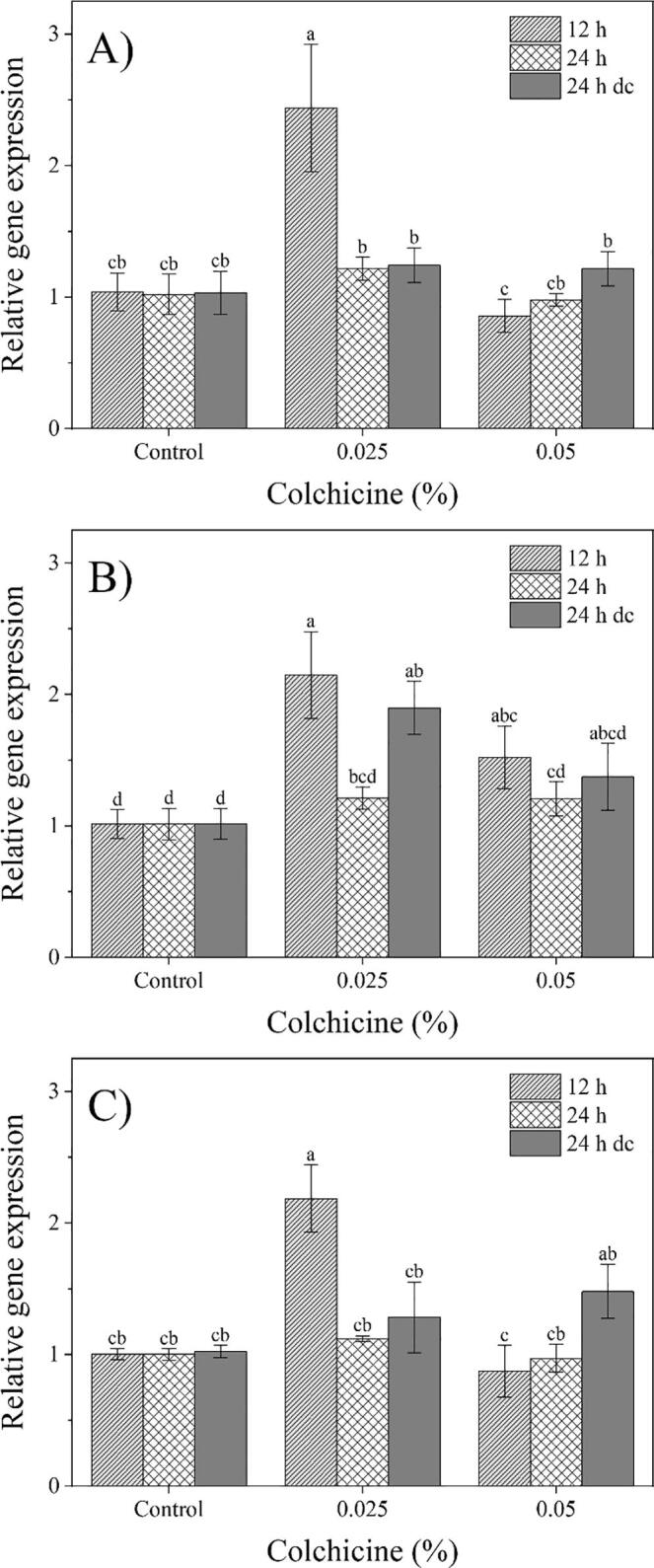
Effect of colchicine on relative expression of apocarotenoids biosynthetic genes in *C. sativus*, including aldehyde dehydrogenase (*ALDH*) (A), β –glucosidase (*BGL*) (B)*,* and carotenoid cleavage dioxygenase 2 (*CCD2*) (C)*.* Error bars are shown as SE (n = 3). Means followed by the same letter are not significantly different (LSD; P ≤ 0.05). 0.05 and 0.025%: Different concentratin of colchicine 12 h and 24 h continuously, and 24 h discontinuously (dc): Different times of plants exposing.

In this paper, the effect of colchicine on the relative genes expression of *CCD2* (carotenoid cleavage dioxygenase), *ALDH* (aldehyde dehydrogenase), and *BGL* (β-glucosidase) involved in the apocarotenoids biosynthesis at the end of the methylerythritol phosphate pathway (MEP) was evaluated (Jain et al., 2016). The results showed a significant increase in gene expression in colchicine-treated plants. The relative expression of *ALDH*, *BGL*, and *CCD2* genes in plants treated with 0.025% colchicine for 12 h was 2-fold higher than the control (Fig. 2). The gene expression of *BGL*, which is involved in the conversion of picrocrocin to safranal in the presence of heat (Jain et al., 2016), exhibited 2 and 1.5 fold increase in treatments with 0.025% colchicine for 24 h dc and 0.05% colchicine for 12 h, respectively, compared with the control (Fig. 2-B). The *CCD2* gene expression in the treatment of 0.05% colchicine for 12 h was 0.8 of the control (Fig. 2-C).

### 3.6 HPLC phytochemical analysis

**Fig. 3:**
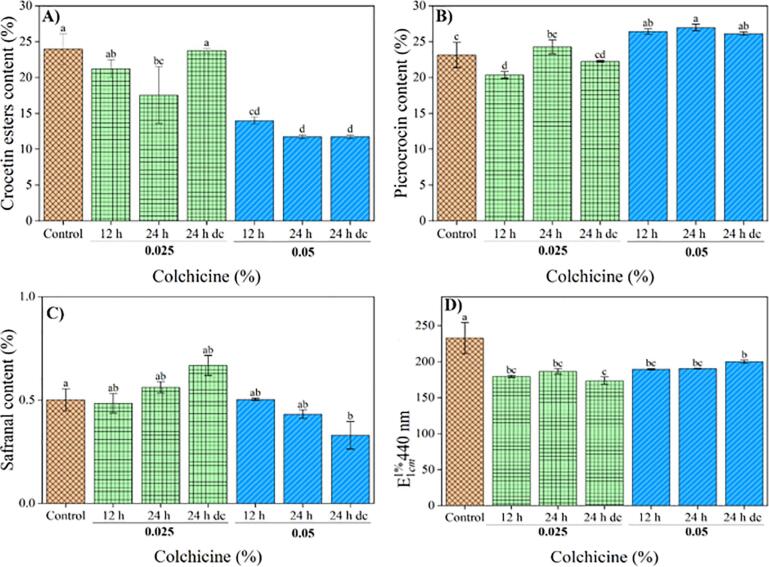
Effect of colchicine on crocetin esters, picrocrocin, safranal and coloring strength content of *C.sativus*. Bar diagram showing crocetin esters (A), picrocrocin (B), safranal (C) and coloring strength (D) content in colchicine treated and control plants. Error bars are shown as SE. Means followed by the same letter are not significantly different (LSD; P ≤ 0.05). 0.05 and 0.025%: Different concentratin of colchicine. 12 h and 24 h continuously, and 24 h discontinuously (dc): Different times of plants exposing.

Stigmas of *C. sativus* were analysed by HPLC at the red stage. The HPLC analyses of the apocarotenoids extracts indicated various peaks with dissimilar contents. The highest crocetin esters concentrations obtained in the control and treatment groups consisted of 0.025% colchicine for 24 h dc and 12 h, respectively. Also, a significant drop in crocetin esters content was observed in 0.05% colchicine in all three-time treatments (Fig. 3**-**A). The highest picrocrocin was recorded in samples treated with 0.05% colchicine in all time treatments, and the lowest in 0.025% colchicine for 12 h (Fig. 3-B). Since treatment with 0.025% colchicine for 24 h dc decreased picrocrocin (Fig. 3-B), a considerable safranal content was observed in this treatment (Fig. 3-C). Also, samples treated with 0.05% colchicine for 24 h dc had the least content of safranal compared with the control (Fig. 3-C).

$E_{1cm}^{1\%}$ 440 nm (colouring strength) is a parameter of stigma colour determined by spectrophotometric analysis ISO 3632 (2011). The colouring strength value is correlated with the ability to transmit colour to the added foodstuff. In this study, based on UV–vis spectrophotometric results, the values of $E_{1cm}^{1\%}$ 440 nm were significantly different in all colchicine samples compared with the control (Fig. 3-D), which was distinct from the results of the crocetin esters content obtained from the HPLC method. The HPLC method allows determining the content of crocetin esters (its concentration), because it is more specific than spectrophotometric UV–vis employed by ISO 3632 (2011), as described by Garcia-Rodriguez et al. (2014). Therefore, the results of crocetin esters obtained from HPLC and the results of colouring strength achieved from UV–vis could be different for control and treatment groups.

## 4 Discussion

*C. sativus* is a main source of crucial medicinal apocarotenoids such as crocetin esters, picrocrocin and safranal. Considering the importance of *C. sativus* from a medicinal and economic perspective, the present study was conducted to identify effective approaches to crop performance improvement, and the content of apocarotenoids. Since *C. sativus* is only grown by corm due to its sterile nature, it is important to create genetic diversity for its quality and quantity improvement. Colchicine, due to the artificial polyploidy induction, and EMS due to the induction of point mutation in the DNA strand, are common chemicals widely used to plant breeding (Rime et al., 2019, Zhou et al., 2017). The goal of this research was to evaluate the effect of colchicine and EMS on the apocarotenoids content of *C. sativus.* However, there are scant reports on gene expression and metabolic alterations of *C. sativus* under colchicine and EMS treatment.

The changes induced by chemical mutagens could be different depending on the plant species, mutagens concentration, and treatment time. On the other, these alterations are probably related to various levels of flexibility in the *C. sativus* plants studied. Zaffar et al. (2003) observed that different colchicine concentrations delayed flower and leaf emergence in *C. sativus*, but there was a reduction in the number of flowers per plant, plant growth rate, and stigma length. By contrast, in the current study, different colchicine treatments had no effect on decreasing the number of flowers in *C. sativus* plant. Moreover, they had no significant effect on the reduction of stigma length. Ali et al. (2012) reported that treatment with 0.25% colchicine had the highest positive effects on morphological characters of *C. sativus*, though some harmful impacts were observed at 1% concentration, and the survival percentage fell at the same concentration. It is worth noting that colchicine can cause different types of mutations, including the deletion or insertion of one or some chromosomes by affecting the genome besides chromosome doubling (Dermen, Smith, & Emsweller, 1943). Additionally, in this research, the growth reduction in some colchicine-treated plants could be attributed to the effect of colchicine on physiological activities and subsequent cell division.

Moreover, Khan et al. (2011) studied the effect of 0.1% EMS and 0.05% colchicine on mother corms, reporting that these chemical mutagens yielded the maximum number of daughter corms in *C. sativus*. On the other, Kashtwari, Wani, Dhar, Jan, and Kamili (2018) observed that the highest weight of daughter cormlets of *C. sativus* was gained under 0.1% EMS treatment. The EMS mutagen could induce point mutations, which lay the basis for mutations like missense and nonsense. As an alkylating agent, EMS can efficiently produce different types of mutations and monitoring these variations is even more challenging than the mutations induced by γ-ray. As a result of this mutagen, the nucleotides match their complementary bases improperly, and this status could induce changes in the sequence of DNA after replication (Watanabe et al., 2007). As discussed in previous studies, these types of diversity in morphological characteristics of *C. sativus* could be attributed to different concentrations of colchicine and EMS mutagens (Salwee & Nehvi, 2017). Further, some of the results are consistent with those reported on other plants including *Gladiolus grandifloras* (Manzoor et al., 2018).

In this study, the colchicine and EMS mutagens affected the features and the number of stigmas in *C. sativus* flowers. Also, different changes induced were probably due to alterations in the genome structure. In the first year, after planting colchicine-treated corms, small and non-tepal flowers emerged. In the second year, different numbers of stigmas including two, four, five, and six threads, as well as deformed flowers or even flowers without stigma were observed (Fig. 1). Zaffar et al. (2003) reported that in the *C. sativus* plants treated with colchicine, there were flowers with irregularly shaped, lobed and dentate tepals as well as reduced number and size of tepal. In the study of Manzoor et al. (2018), at 0.1% colchicine treatments, there were flower petals with serrated margins as well as flowers with oval-shaped petals at 0.2% colchicine in *G. grandiflorus* plants. Besides, pointed outgrowth on petals and surface were observed in both concentrations, respectively.

Colchicine can bind to microtubules and prevent the orientation and movement of chromosomes to the opposite pole during the mitosis and halt the cell cycle (Eng & Ho, 2019). Colchicine can have varying effects on plant cells, which is probably exacerbated if the treated plants are in their growing season, and cells are dividing. In other words, different chromosomal changes may occur in the genome structure (Dermen et al., 1943). That is, genetic modification using colchicine would probably produce some new plant cultivars by changing the plant genetic structure (Obute, Ndukwu, & Chukwu, 2007).

Further, EMS had severe effects on *C. sativus* corms and flowers, and most of the corms did not sprout due to disintegration and crushing (Supplementary File 1. Figure S5). According to the results, the destructive changes in most of the corm’s tissues explained their failed sprouting. This could be attributed to the high production rate of mutations by EMS (Chen et al., 2018), which led to the biological and metabolic disruptions of corms and ultimately their death (Supplementary File 1. Figure S5). Besides, EMS-treated plants triggered different changes in the *C. sativus* flowers, such as asymmetric tepals and incomplete stigmas (Supplementary File 1. Figure S6). Compared with colchicine, the harmful effects of EMS were so severe that most of the EMS-treated corms had not grown properly or survived in the first and second year of the experiment.

It is important to note that different changes induced in the first and second years may be due to possible epigenetic changes. There is limited known about *C. sativus* epigenetics, but we know that it changes from one growing season to another and remains stable under natural conditions (Busconi et al., 2018). Also, environmental factors and physiological conditions during the plant growth period should be considered in the evaluation of changes (El-Nashar & Asrar, 2016).

The results of flow cytometry did not show any significant change in the genome size of *C. sativus* plants treated with colchicine (Supplementary File 2. Table S2). Many chemical mutagens, as inhibitors, affect the plant genome structure. For instance, colchicine can block the cell division cycle in plant cells, and mutagen-induced changes depend on the type of plant species (Manzoor et al., 2019). Thus, sometimes it is not easy to predict the effect of colchicine as a mutagen agent on the plant chromosome (Dermen et al., 1943).

Previous research has only studied the effects of mutagens on morphological characteristics of *C. sativus* plants. In this study, we focused on the effect of colchicine and EMS mutagens on important secondary metabolites of *C. sativus*. Thus, to gain deeper insights into the mechanism by which colchicine changes the secondary metabolic pathways, we investigated the expression of genes involved in the key pathway of specialized metabolites such as crocetin esters, safranal and picrocrocin. As mentioned earlier, since most of the EMS-treated plants died after exposure and their evaluation was not possible, we only assessed colchicine-treated plants.

The biosynthesis pathway of the *C. sativus* apocarotenoids, which makes it an important crop (Wani et al., 2017), begins by the oxidative cleavage of zeaxanthin in several steps, which are catalysed by the carotenoid cleavage dioxygenase (CCD2), aldehyde dehydrogenase (ALDH) and UDP-glycosyltransferases (UGTs) enzymes. Finally, due to the combined effect of heat and β-glucosidase (BGL), picrocrocin is transformed into safranal (Ahrazem et al., 2016, Frusciante et al., 2014, Jain et al., 2016). According to RNA-seq data, the analysis of expression patterns of transcripts involved in key enzymatic steps of apocarotenoids biosynthesis, including carotenoid cleavage dioxygenase, glucosyltransferases, aldehyde dehydrogenases, and beta glucosidases, demonstrate their higher expression levels in stigma than in other tissues (Jain et al., 2016). Furthermore, the highest *ALDH*, *BGL*, and *CCD2* genes relative expression appeared at the red stage of stigma (Frusciante et al., 2014). Thus, to evaluate the effect of colchicine mutagen on *ALDH*, *BGL*, and *CCD2* genes relative expression as well as apocarotenoids content, all flower stigmas were collected at the red stage (before anthesis).

Colchicine is recognized as one of the most common mutagens used for polyploidy. However, its mechanism has not been adequately studied in different plants and studies have mainly focused on human health (Zhou et al., 2017). Likewise, as a sterile plant, one of the main challenges facing *C. sativus* breeding programs is polyploidization by colchicine. Each plant species gives various responses to polyploidy based on its genomic structure, ploidy level, and reproduction patterns (Manzoor et al., 2019), and the effects of this method on plant metabolism are still unclear (Tan et al., 2017). Also, it is not easy to predict alterations caused by colchicine and it might even provoke a wide range of changes (Dermen et al., 1943). These are challenges that complicate the description of changes in the expression pattern of key genes, and consequently apocarotenoids biosynthesis pathway.

The highest relative expression of *ALDH*, *BGL*, and *CCD2* genes in stigma was observed in 0.025% colchicine treatment for 12 h. Hence, the results revealed that the contents of crocetin esters, safranal, and picrocrocin of *C. sativus* are influenced by colchicine at the transcript level. On the contrary, the apocarotenoids content was also variable. That is, the increase or decrease in crocetin esters, picrocrocin and safranal content did not represent an exactly equal trend, which may be due to disturbance induced by colchicine in the metabolic pathway that controls the biosynthesis of compounds following transcript regulation.

The gene expression regulation in plants is influenced by several biological processes, including environmental stresses, the balance of metabolic and physiological pathways, the process of growth and development, and so forth. On the other hand, the synthesis and accumulation of secondary metabolites in plants like medicinal herbs are complicated due to the internal developmental genetic flows (different enzymes and regulated genes) and external environment factors (climate, light, temperature, water, salinity, etc.) (Li, Kong, Fu, Sussman, & Wu, 2020). Thus, all of changes that influence genes expression followed by the production of secondary metabolites in plants may be due to alteration in normal cell activities and phytohormones involvement under various factors. It would lead to different levels of gene expression, and consequently different contents of the secondary metabolites (Wani et al., 2017, Zhou et al., 2017). Thus, when plant cells are challenged by mutagen such as colchicine, the final chemical productions may fluctuate depending on the type of plant species and their internal and external growing conditions. Therefore, the unpredictable responses elicited by different treatments of colchicine could be the result of factors that alter the gene expression and protein metabolism trend.

Previous studies have reported divergent results about the alteration of secondary metabolite content by colchicine in other plants. For example, Noori, Norouzi, Karimzadeh, Shirkool, and Niazian (2017) reported that the thymol content of *Trachyspermum ammi,* as the main secondary metabolite in tetraploid plants, was greater than diploid plants. Thus, the results of the current study suggest that, despite the absence of polyploidy, different treatments might have directed metabolic flow towards a drop or surge in the production of apocarotenoids of crocetin esters, picrocrocin, and safranal. Thus, there was a fluctuating trend in the specialized metabolites biosynthesis pathway.

In general, it is worth noting that plant cells are somehow resistant to colchicine. The relative resistance of plant microtubule polymerization to colchicine originates from the low affinity of colchicine and tubulin. The biochemical mechanism of this resistance is not completely known, and different plant species will probably display dissimilar reactions (Morejohn, Bureau, Tocchi, & Fosket, 1987). It also appears that in the current study, colchicine is faced with a molecular resistance in the plants so that it could not cause so many changes. Thus, although colchicine can affect the metabolic and morphology of herbs, its responding process is not predictable in plants with varying levels of genome and life cycles. Consequently, identifying mutations and monitoring the induced changes in the genome is usually a challenging way, in particular, in such plants as saffron with the triploid genome. So one of the more important aspects of the current study was to investigate the more available ways to recognize the probable changes that were expected induced by the mutagens. On the other hand, the other advantage is that this has been for the first time that the research on the effect of mutagens on gene expression and secondary metabolites content has been done and can be considered as a basic research for other studies in this field in the future. In addition, it is necessary to mention, the offspring of the colchicine-treated plants was checked, but actually, none of the next generations showed different behaviors. In fact, in the third year, we even did the flow cytometry test to estimate the genome size of the progeny of the treated plants that in the second year had flowers with different numbers of stigma compared with the control but the size of the genome was not changed. Accordingly, at first, for the successful implementation of the plant breeding programs in the future coming, it is recommended to experiment on these mutagens at different concentrations and times. Secondly, since through the method tested, induce polyploidy could not happen and identifying other probable genetic mutations in each plant in the next generation was not enforceable separately, more studies are needed to be carried out to choose the better way to induce polyploidy in *C. sativus* as well as check the offspring individually in terms of other probable mutations in the future. In addition, as a suggestion, since RNA sequencing is a powerful technique for profiling the complete gene can be done to investigate the progeny in the next studies after the mutagen applications because, in this way, the offspring can be individually identified and assayed. Moreover, understanding dynamic and stable variations in *C. sativus* plants is a subject that could be pursued in future research.

## 5 Conclusion

The aim of this study was to investigate the effects of colchicine and EMS mutagens on different characteristics of the *C. sativus* plant. The results suggested that mutagens could produce various effects on the morphology and cellular metabolism of *C. sativus*. The analysis also revealed that the *C. sativus* plants could demonstrate varied reactions to colchicine and EMS mutagens depending on their distinct traits. As shown by the evidence, the positive effect of colchicine outweighed that of EMS. The highest expression level of genes involved in apocarotenoids production (*ALDH*, *BGL*, and *CCD2*) in *C. sativus* was observed under 0.025% colchicine treatment for 12 h. However, the HPLC analysis of the apocarotenoids indicated that the content of secondary metabolites varied in different colchicine treatments. The changes could be attributed to the metabolites decomposition in their synthesis pathway or the shifting of direction to produce other secondary metabolisms as a result of possible mutations. The findings suggest that colchicine is an effective mutagen that could be applied to breeding methods of *C. sativus.* Accordingly, the above treatments would probably pave the way for producing changes in *C. sativus*. However, further research is warranted to explore genes that regulate the secondary metabolites biosynthesis pathway of *C. sativus*.

## Declaration of Competing Interest

The authors declare that they have no known competing financial interests or personal relationships that could have appeared to influence the work reported in this paper.
