# Supplementary material for: Morphological, molecular and phytochemical variations induced by colchicine and EMS chemical mutagens in Crocus sativus L
Source: Food Chem (Oxf). 2022 Feb 14;4:100086. doi: 10.1016/j.fochms.2022.100086 (PMC8991884; doi:10.1016/j.fochms.2022.100086)

**Supplementary Figures**

**Figure S1**. A microscopic image of *C. sativus* stigma with 100X magnitude (Zeiss Co., Model: Axiophot, Germany) (A) and Summary of schematic representation of the gene expression of enzymes involved in apocarotenoid biosynthetic pathway in *C. sativus* (B).

**
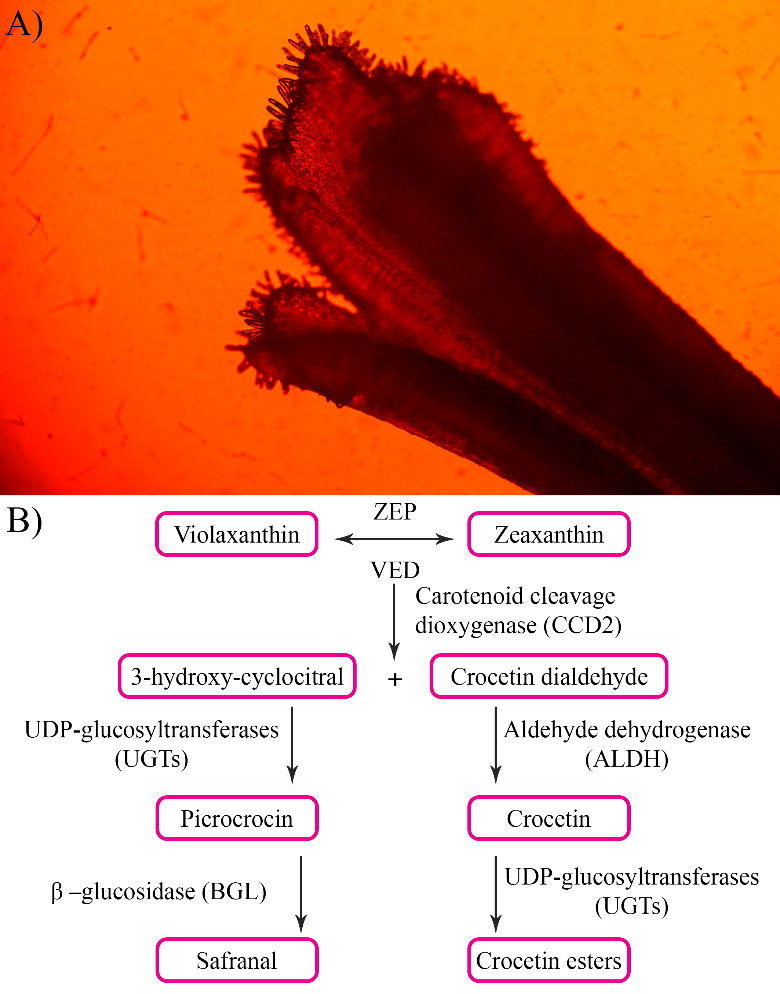
**

**Figure S2.** Agarose gel electrophoresis of RNA extracted from treated *Crocus sativus* L*.*

**
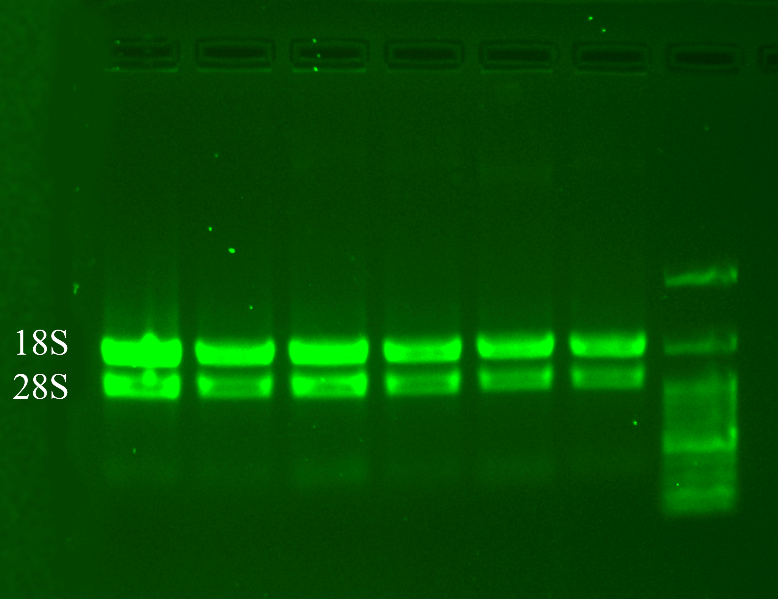
**

**Figure S3.** The amplification *ALDH* (A), *BGL* (C), *CCD2* (E) and *18S* (G) The melting curves of amplicons of *ALDH* (B), *BGL* (D), *CCD2* (F), *18S* (H).


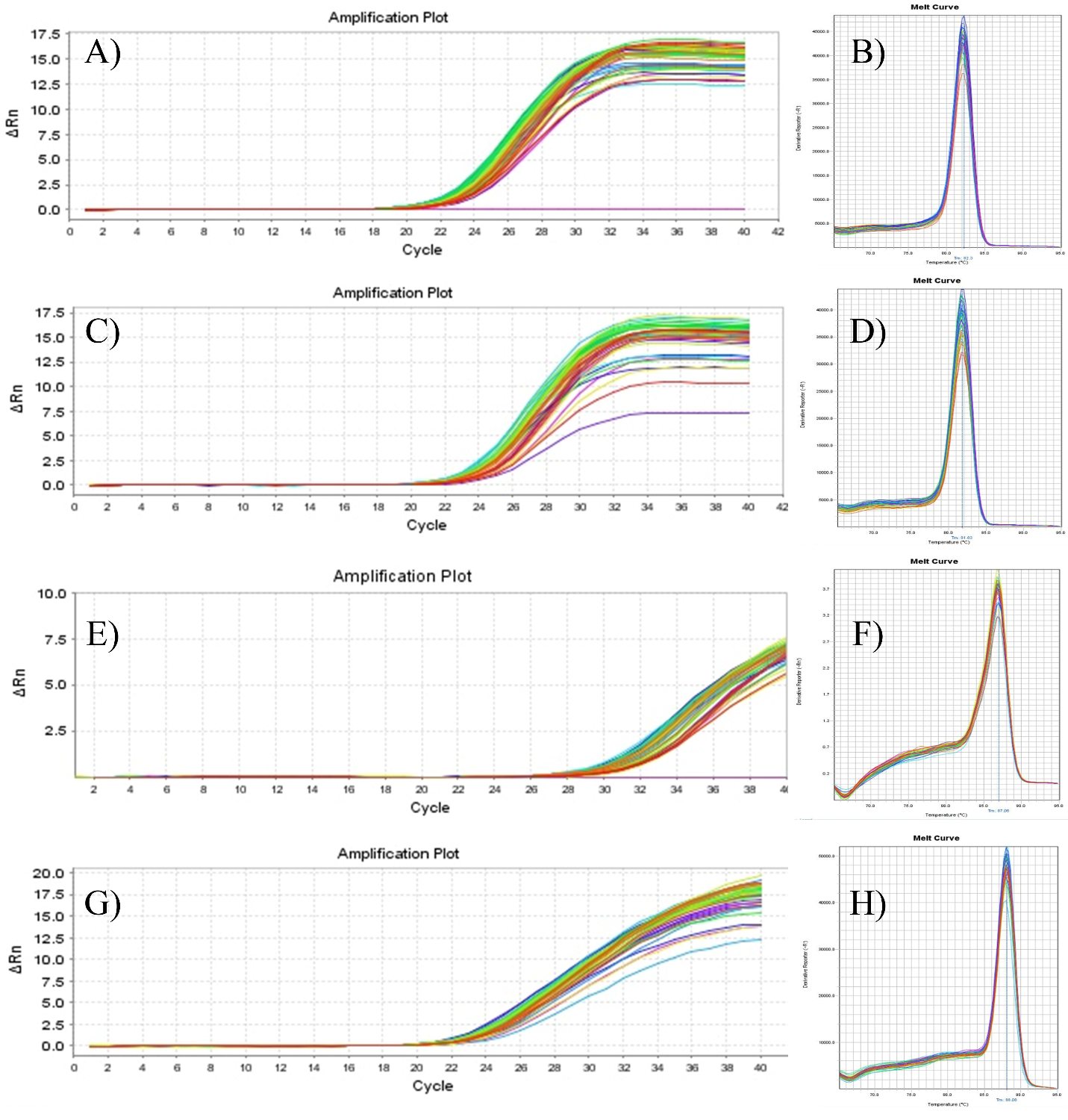


**Figure S4.** Effect of colchicine on flowers shapes of *C. sativus*. Flowers without tepals (A) and stigma (B), and control (C).


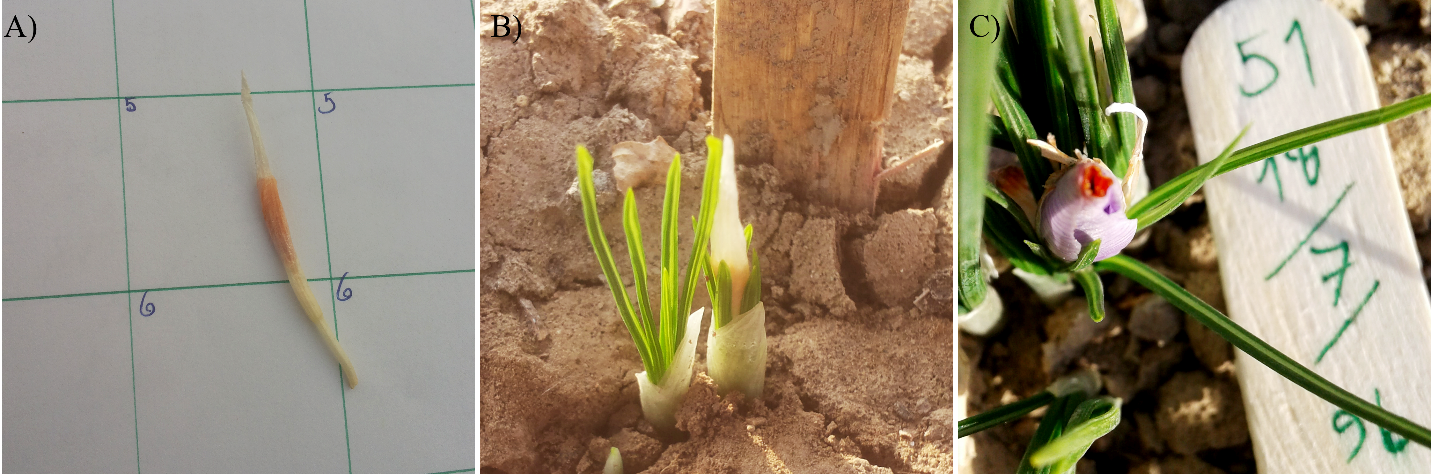


**Figure S5.** Effects of EMS on corms of *C. sativus*. A disintegrated corm (A), a crushed corm (B), and controls (C) and (D).


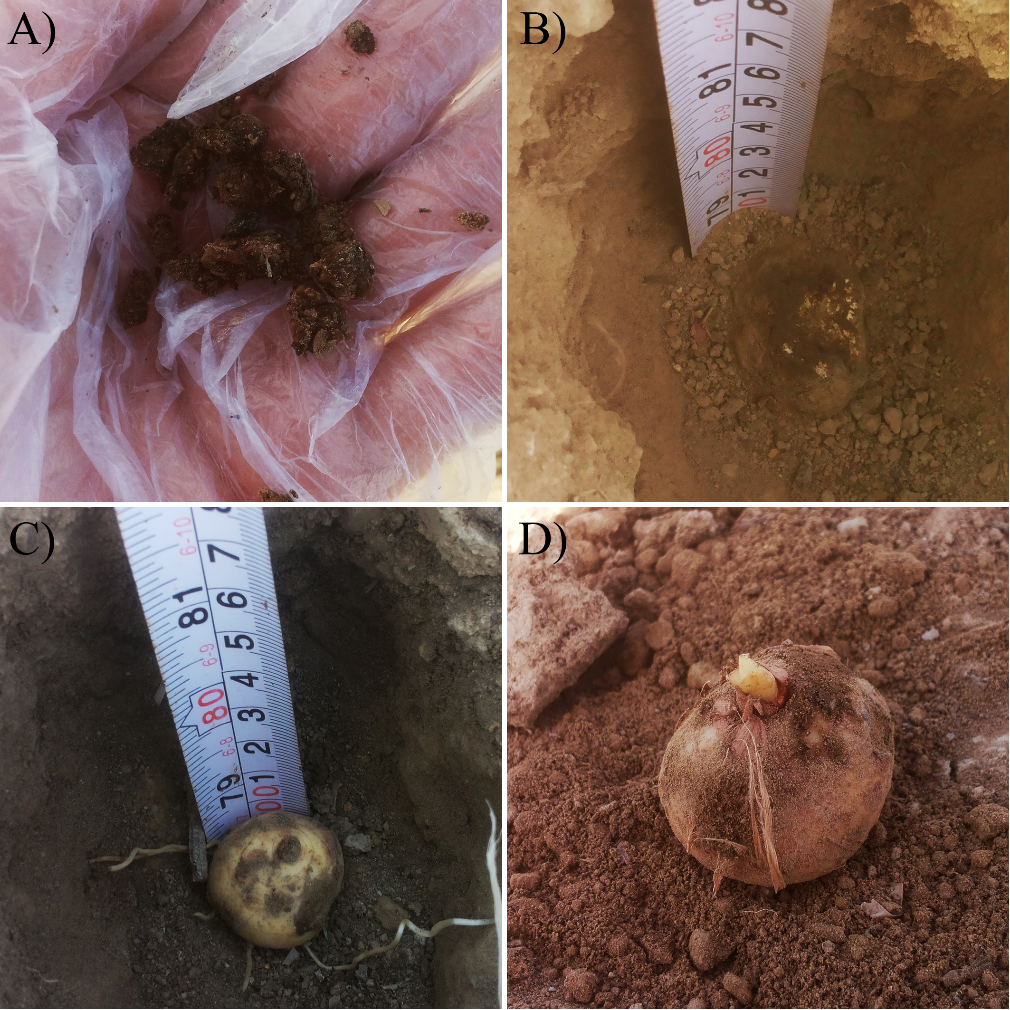


i1

**Figure S6**. Effect of EMS on flowers shape and stigma of *C. sativus*. Control (A), lack of symmetry in tepals (B), irregular flower and angled tepal (C), and an incomplete stigma (D).


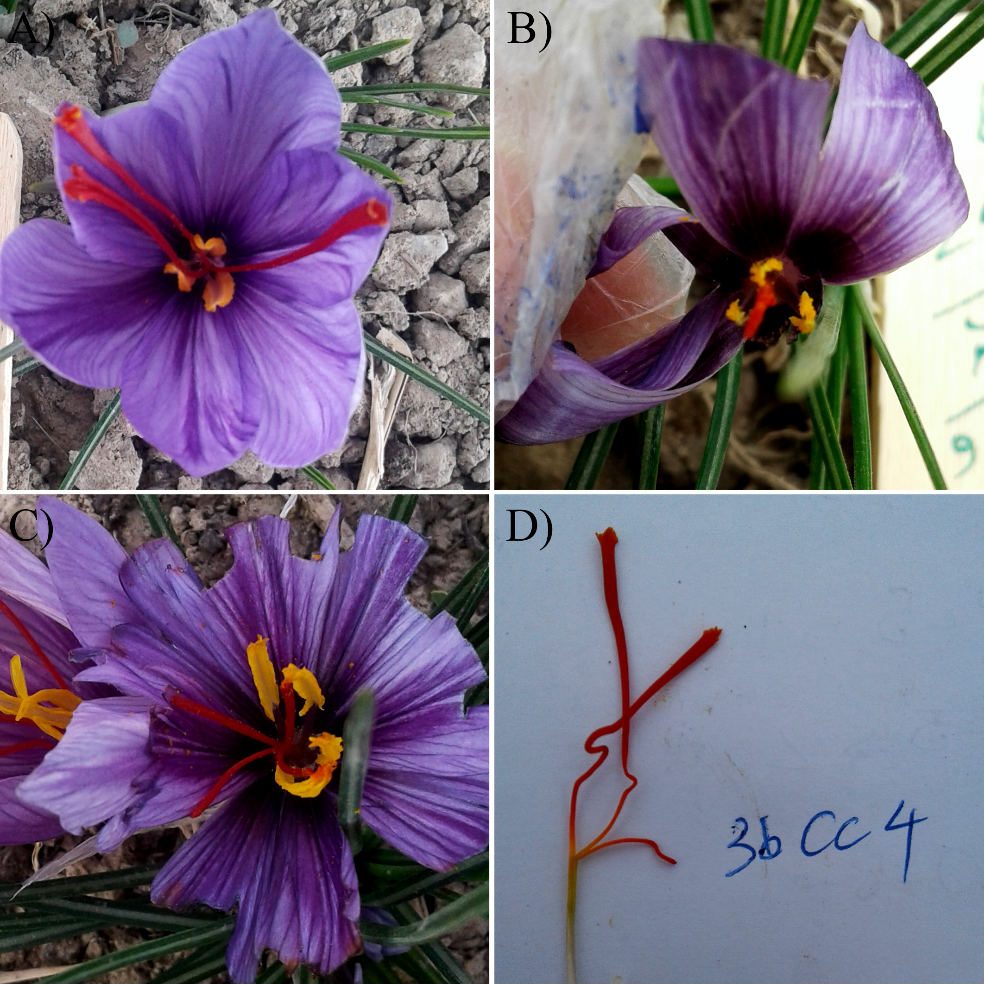


**Figure S7**. Histograms of flow cytometric 2C DNA content and somatic chromosomes of *C. sativus* (1). Colchicicne treatmes including 0.05% + 24 h continuously (A), 0.05% + 24 h discontinuously (dc) (B), 0.05% + 12 h continuously (C), 0.025% + 24 h continuously (D), 0.025% + 24 h discontinuously (dc) (E), 0.025% +12 h continuously (F), Control (G). *Vicia faba* cv. Inovec was used as an internal standard (2).


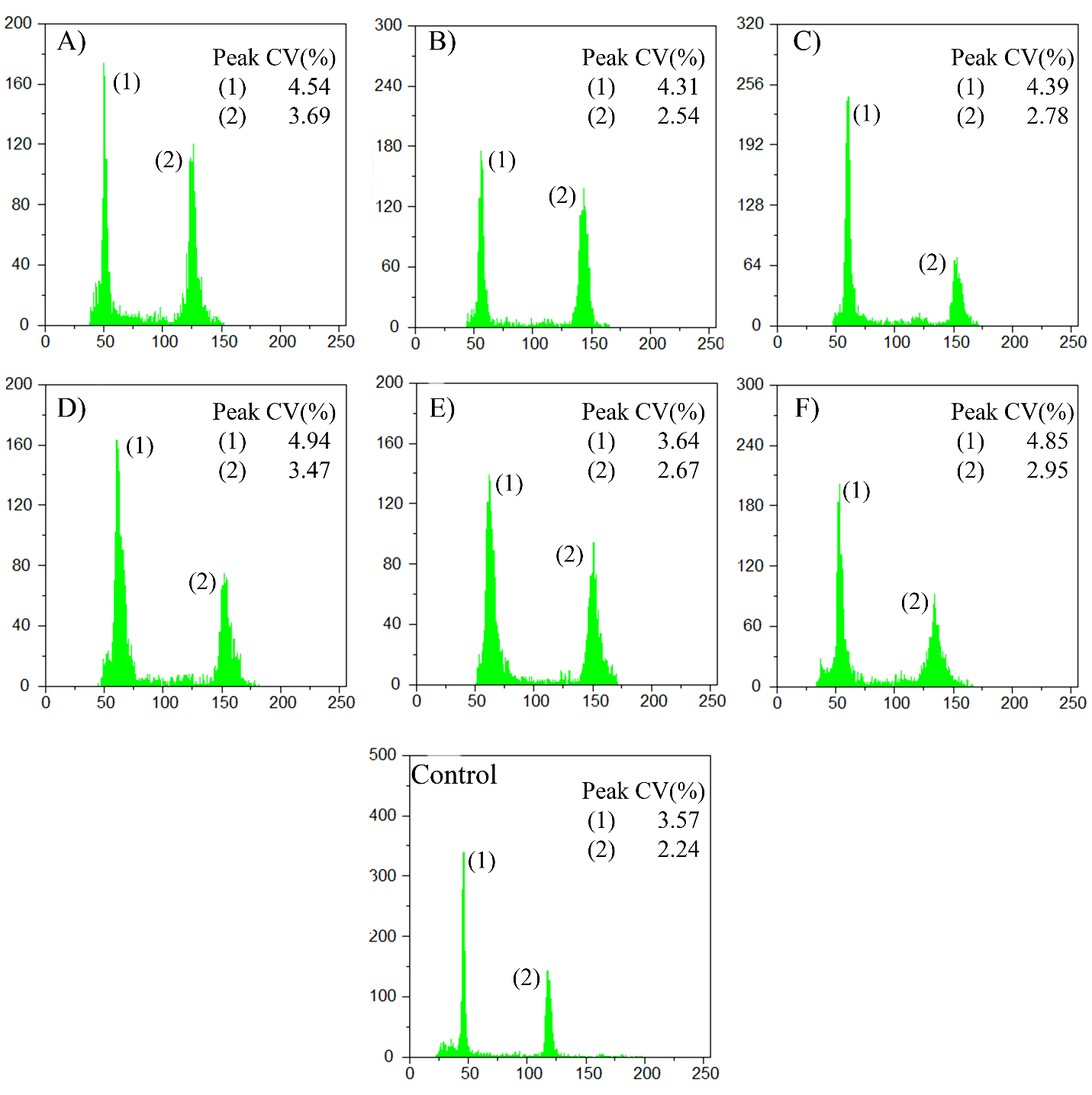

Supplement: Supplementary data 1 [file mmc1.docx]
