# Supplementary material for: Morphological, molecular and phytochemical variations induced by colchicine and EMS chemical mutagens in Crocus sativus L
Source: Food Chem (Oxf). 2022 Feb 14;4:100086. doi: 10.1016/j.fochms.2022.100086 (PMC8991884; doi:10.1016/j.fochms.2022.100086)
Supplement: Supplementary data 2 [file mmc2.docx]

**Supplementary Tables**

**Table S1.** The sequences of Primers used in qPCR.

| Genes | accession number (Gene Bank) | Forward and Reverse Primer Sequences | Fragment size (bp) |
| --- | --- | --- | --- |
| *ALDH*    *BGL*  *CCD2*  *18S* | KU577904  KX790358  KP887110  AB699586 | F: 5ʹ-AAGGTGCCAGCTCAACTTAC-3ʹ  R: 5ʹ-CTCCTATGACTGGTTCGAGAGA-3ʹ    F: 5ʹ-ACGCAAGTATCAGGCTTCTC-3ʹ  R: 5ʹ-GACAGCATCCATATCCCTCTTG-3ʹ    F: 5ʹ-CATTACTACCTCTCCGGCAATTA-3ʹ  R: 5ʹ-AGGACCAACTCTCAGAAACAC-3ʹ    F: 5ʹ-ATCATTGTCGAGACCCGAAC-3ʹ  R: 5ʹ-TCGTTTCCTCCAAAATCTCG-3ʹ | 127  100  120  119 |

| Colchicine treatment | | Ploidy  Level 2n 2C DNA mean  (pg) ± SE | | | Monoploid | |
| --- | --- | --- | --- | --- | --- | --- |
|  |  |  |  |  | (1C_X_ DNA)  genome size (pg) | (1C_X_ DNA)  genome size (Mbp) |
| 0.05% | 24 h | 3_X_ 24 | | 10.91 ± 0.07 | 3.636 | 3556.00 |
|  | 24 h dc | 3_X_ | 24 | 10.75 ± 0.10 | 3.583 | 3504.17 |
|  | 12 h | 3_X_ | 24 | 10.77 ± 0.10 | 3.590 | 3511.02 |
| 0.025% | 24 h | 3_X_ | 24 | 11.03 ± 0.10 | 3.676 | 3595.12 |
|  | 24 h dc | 3_X_ | 24 | 10.60 ± 0.08 | 3.533 | 3455.27 |
|  | 24 h | 3_X_ | 24 | 10.88 ± 0.30 | 3.626 | 3546.22 |
| 0.00% | Control | 3_X_ | 24 | 10.68 ± 0.25 | 3.560 | 3481.68 |

**Table S2.** Effect of colchicine on genome size of *C.sativus.* Values represented Mean ± SE

*C. sativus* plants following exposure to colchicine mutagen (0.05 and 0.025%) for 12 h and 24 h continuously, and 24 h discontinuously (dc).
